# pH-Dependent Metal Ion Toxicity Influences the Antibacterial Activity of Two Natural Mineral Mixtures

**DOI:** 10.1371/journal.pone.0009456

**Published:** 2010-03-01

**Authors:** Tanya M. Cunningham, Jennifer L. Koehl, Jack S. Summers, Shelley E. Haydel

**Affiliations:** 1 School of Life Sciences, Arizona State University, Tempe, Arizona, United States of America; 2 The Biodesign Institute Center for Infectious Diseases and Vaccinology, Arizona State University, Tempe, Arizona, United States of America; 3 Department of Chemistry, Western Carolina University, Cullowhee, North Carolina, United States of America; Baylor College of Medicine, United States of America

## Abstract

**Background:**

Recent studies have demonstrated that several mineral products sold for medicinal purposes demonstrate antimicrobial activity, but little is known about the physicochemical properties involved in antibacterial activity.

**Methodology/Principal Findings:**

Using *in vitro* mineral suspension testing, we have identified two natural mineral mixtures, arbitrarily designated BY07 and CB07, with antibacterial activity against a broad-spectrum of bacterial pathogens. Mineral-derived aqueous leachates also exhibited antibacterial activity, revealing that chemical, not physical, mineral characteristics were responsible for the observed activity. The chemical properties essential for bactericidal activity against *Escherichia coli* were probed by testing antibacterial activity in the presence of metal chelators, the hydroxyl radical scavenger, thiourea, and varying pH levels. Chelation of the BY07 minerals with EDTA or desferrioxamine eliminated or reduced BY07 toxicity, respectively, suggesting a role of an acid-soluble metal species, particularly Fe^3+^ or other sequestered metal cations, in mineral toxicity. This conclusion was supported by NMR relaxation data, which indicated that BY07 and CB07 leachates contained higher concentrations of chemically accessible metal ions than leachates from non-bactericidal mineral samples.

**Conclusions/Significance:**

We conclude that the acidic environment of the hydrated minerals significantly contributes to antibacterial activity by increasing the availability and toxicity of metal ions. These findings provide impetus for further investigation of the physiological effects of mineral products and their applications in complementary antibacterial therapies.

## Introduction

Over 80 years ago, the first commercial antibiotic, penicillin, was identified and the “antibiotic era” was launched. As new antibiotics were developed and administered to patients over the subsequent decades, modern medicine significantly improved, deadly infections were effectively treated, and life expectancy increased dramatically. However, our arsenal of antimicrobials is currently under attack by the microorganisms themselves as clinically-significant, antibiotic-resistant bacteria evolve at alarming rates. In addition, there is an ongoing need to develop therapeutic treatments for bacterial infections, which are not curable by currently-available antibiotics, such as advanced Buruli ulcer disease.

Medicinal and therapeutic use of mineral products has impacted human health throughout recorded history. Early research focused on the extraordinary absorptive and adsorptive physical properties of clay minerals and mineral mixtures and the health benefits recognized in aiding digestive processes [1], cleansing and protecting the skin [2], and serving as substitute sources of essential minerals [3], [4]. The healing effects of clay minerals have primarily been attributed to physical properties, whereby the layered structure and charged surfaces generate a large surface area allowing adsorption of toxins, metals, and oils from the skin or digestive tract [2], [5].

Because clay mineral surfaces are charged, molecules such as proteins, nucleic acids, and cations bound to water can be adsorbed onto the clay surface in an aqueous suspension [6]. Clays with expandable interlayers can also absorb hydrated cations within the layers. When clays are hydrated, adsorbed and absorbed ionic species can be exchanged dependent on the concentration in the solution and cation selectivity of the clay [5]. Successful synthetic production of bactericidal mineral products by Ag^+^- and Cu^2+^-cation exchange has been reported [7], [8], [9]. Transition metals such as iron, copper, aluminum, and zinc are commonly found in exchangeable sites and are known to have toxic effects on bacteria either by competing with essential enzyme cofactors, by irreversibly binding biological molecules to inhibit function, or by replacing ions essential to membrane stabilization [10]. In addition, metal cations, such as iron and copper, have been implicated in production of hydroxyl radicals and DNA damage in the cell [11], [12]. Among mineral products sold for health purposes in African markets, geological mixtures with the strongest antibacterial activity were primarily composed of kaolinite [13], which has been demonstrated to cause degradation of nucleic acids *in vitro* [14]. Recently, the antibacterial properties of red soils from Jordan were attributed to biotic factors, whereby antimicrobials were produced by proliferating mineral-associated bacteria [15]. To our knowledge, the role of physicochemical properties in antimicrobial activity has not yet been investigated for any of the bactericidal mineral products identified.

Documented use of two clay minerals as a therapeutic treatment for the necrotizing *Mycobacterium ulcerans* skin infection, Buruli ulcer, suggests that specific natural mineral products have significant beneficial effects on wound healing [16]. We have previously demonstrated that one of two mineral products used to heal Buruli ulcer infections [16] has antibacterial activity against a broad-spectrum of pathogenic bacteria [17], suggesting the potential for therapeutic applications of minerals against topical infections. We have screened approximately 50 mineral mixtures sold as health and cosmetic products and identified two additional mineral mixtures with antibacterial activity, indicating that antibacterial minerals are diverse and that the associated health benefits may be related to bactericidal activity.

Here, we report broad-spectrum antibacterial activity of two mineral mixtures, arbitrarily designated BY07 and CB07, and their aqueous leachate derivatives. The BY07 and CB07 products are composed of heterogeneous minerals (Table S1) and are therefore referred to as mineral mixtures. We also describe the physicochemical properties essential for antibacterial activity through the application of metal chelators and targeted alteration of pH conditions of the aqueous mineral suspensions and derivatives.

## Results

### Broad-Spectrum Antibacterial Activity

**Figure 1:**
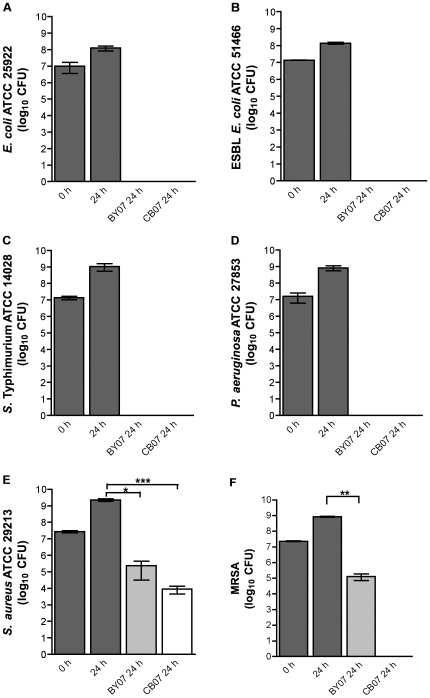
Antibacterial activity of BY07 and CB07 mineral mixtures against antibiotic-susceptible and antibiotic-resistant bacteria. Values represent the mean CFU/400 µL and SD from at least three independent experiments. Statistical significance (paired t-test) of mineral-inoculated 24 h bacterial growth compared to standard 24 h growth: *** *p*<0.0001; ** *p* = 0.0018; * *p* = 0.0179.

The effects of the BY07 and CB07 mineral mixtures on the growth of *E. coli*, extended-spectrum beta-lactamase (ESBL) *E. coli*, *Pseudomonas aeruginosa*, *Salmonella enterica* serovar Typhimurium, *Staphylococcus aureus*, and methicillin-resistant *S. aureus* (MRSA), were investigated by performing *in vitro* antimicrobial susceptibility experiments in broth cultures. Bacteria (initial concentrations of ∼10^7^ CFU) were incubated in BY07 or CB07 mineral suspensions in the appropriate bacterial culture media for 24 h prior to plating to determine viability. Incubations with either concentrated BY07 or CB07 suspensions completely eliminated *E. coli*, ESBL *E. coli*, *P. aeruginosa*, and *S.* Typhimurium viability within 24 h (Figure 1). Incubation of antibiotic-susceptible *S. aureus* with the BY07 and CB07 mineral suspensions resulted in 3-log_10_ and 5-log_10_ reductions, respectively (Figure 1). MRSA viability was reduced 10,000-fold upon suspension in BY07 and was completely eliminated in the presence of the CB07 mineral suspension (Figure 1).

### Chemical and Mineralogical Composition

X-ray diffraction analysis revealed that the most abundant minerals present in BY07 are Ca-smectite clay (37.3%), followed by anorthoclase feldspar (23.0%) and quartz (13.7%) (Table S1). CB07 is a mineral mixture composed primarily of quartz (45.5%), illite (19.8%), and Ca-smectite (17.2%) (Table S1). Quartz and feldspar are tetrahedral silicate minerals and represent two of the most common minerals in the earth's crust [18]. Smectites (calcium and ferruginous), composing 37.3% of BY07 and 21.4% of CB07, are composed of a layered structure of two silicate tetrahedra and one octahedra forming 10 Å separated sheets with an expandable interlayer [19]. Smectite has a high cation exchange capacity, such that when the clay is in a solution saturated with a particular cation, the interlayer cations will be replaced by the cation in excess [20]. Illite has the same structure, but an alternate ionic composition results in a charge imbalance between the sheets, which strongly binds the mineral layers together [5]. Both clay and non-clay mineral surfaces can adsorb bacteria and viruses via electrostatic and hydrophobic interactions [21]. Adsorption of viruses to clay surfaces can be reduced by adhesion of proteins to block negatively charged exchangeable sites [22].

**Figure 2:**
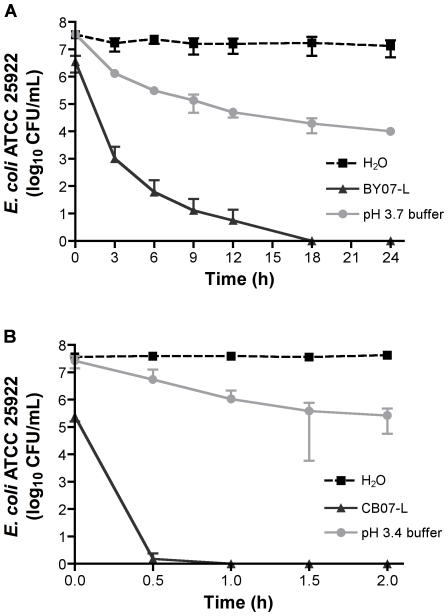
*E. coli* survival in mineral leachates and low pH phosphate buffers. *E. coli* survival in BY07 leachate (BY07-L) and pH 3.7 sodium phosphate buffer for 24 h (A) and CB07 leachate (CB07-L) and pH 3.4 sodium phosphate buffer for 2 h (B). Values represent the mean CFU and SD of at least three independent experiments.

To determine if the mineral mixtures were killing *E. coli* via physical or chemical interactions, aqueous mineral leachates were prepared as described in the Methods section. Suspension of early logarithmic phase *E. coli* in the BY07 and CB07 mineral leachate solutions, devoid of physical minerals, for 24 h completely eliminated bacterial viability (Figure 2). The retention of antibacterial activity in aqueous leachates after removal of mineral particles suggests that the mechanism of antibacterial activity is dependent on soluble molecules and ions released from the minerals upon hydration, rather than physical shearing or electrostatic interactions between mineral and bacterial surfaces. Babich and Stotzky [23] demonstrated that homoionic minerals could desorb metal ions via exchange with ions in the medium and inhibit bacterial growth. Conversely, addition of clay minerals with heterogeneous exchangeable cations including Ca^2+^, Mg^2+^, Na^+^, and K^+^ was shown to reduce toxicity of metals *in vitro* by adsorption of metal ions from the medium [23].

X-ray fluorescence (XRF) analysis of the major chemical components of the BY07 and CB07 mineral mixtures expectedly revealed that silica and aluminum, which compose the crystal structure of smectites and quartz minerals, were the predominant elements present (Table S2). Ca^2+^, Mg^2+^, Na^+^, and K^+^ ions are essential to bacterial cells [10] and are therefore unlikely to contribute to antibacterial activity. TiO_2_ was also present in all samples analyzed and is commonly found on clay and non-clay mineral surfaces. TiO_2_ irreversibly adsorbs *E. coli* via van der Waals and acid-base attractions occurring between lipopolysaccharide O antigens and the metal oxide [24]. TiO_2_ has been shown to inhibit *E. coli* growth only at levels above 360 ppm in media [25], [26]. The concentration of TiO_2_ in the BY07 and CB07 mineral mixtures and leachates is too low to gauge the toxic potential from the XRF data.

### Chelator Treatment and pH Adjustment

**Figure 3:**
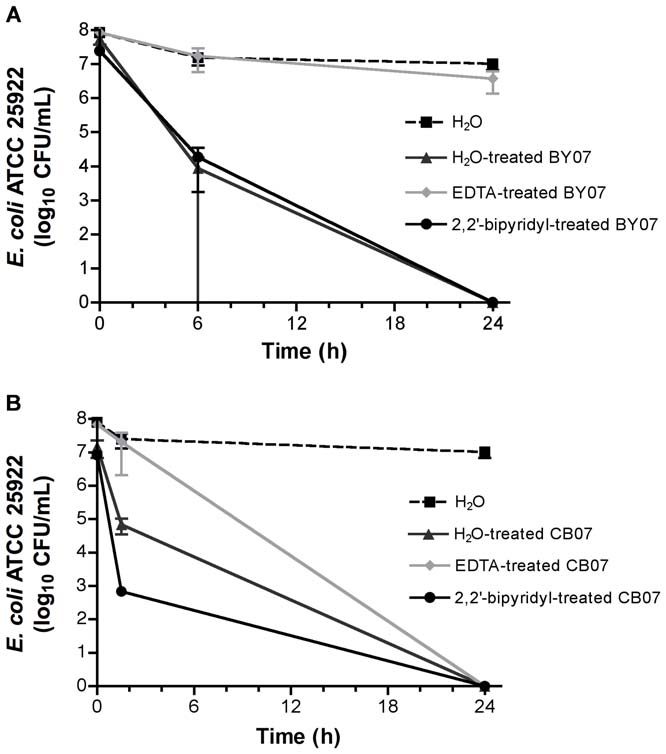
*E. coli* survival in 10% mineral suspensions pre-treated with dH_2_O, EDTA, or 2,2′-bipyridyl. *E. coli* were incubated in 10% suspensions (100 mg/mL) of BY07 (A) and CB07 (B) minerals that had been pre-treated with dH_2_O, EDTA, or 2,2′-bipyridyl. Values represent the mean CFU and SD of three independent experiments.

**Figure 4:**
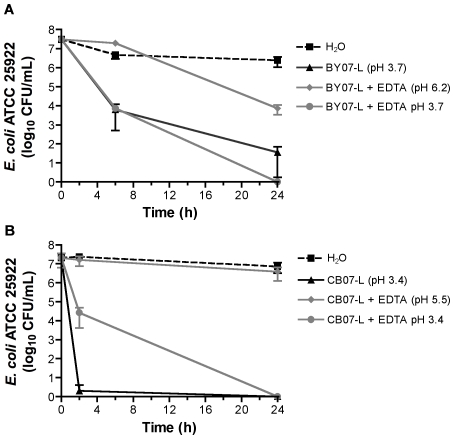
*E. coli* survival in mineral leachates with EDTA and pH adjustment. Survival of *E. coli* in BY07 (A) and CB07 (B) with and without addition of 10 mM EDTA (final concentration) and adjustment of pH. pH values shown within parentheses represent unadjusted pH readings. Values represent the mean CFU and SD of at least three independent experiments.

Because minerals can exchange or leach potentially toxic metal ions from surface sites, the impact of divalent and trivalent cations on antibacterial activity was assessed by pre-treatment of BY07 and CB07 minerals with the broad-spectrum chelator, ethylenediaminetetraacetic acid di-sodium salt (EDTA). Pre-washing of the mineral mixtures with EDTA did not cause a notable change in the major chemical composition of BY07 and CB07 (Table S2). The divalent and trivalent cations most avidly chelated by EDTA are expected to be present at trace levels and are therefore not detected in the XRF analysis. Pre-washing of the BY07 minerals with 100 mM EDTA eliminated BY07 antibacterial activity against *E. coli* (Figure 3). Although antibacterial activity of CB07 pre-washed with EDTA was reduced at 1.5 h, relative to CB07 washed with water alone, both water- and EDTA-washed CB07 resulted in complete bacterial killing at 24 h (Figure 3). When 10 mM EDTA was added directly to the aqueous mineral leachate solutions, bactericidal activity was eliminated in both BY07 and CB07 leachates (Figure 4). The addition of 1 mM and 5 mM EDTA directly to the BY07 and CB07 leachates also resulted in an increase in bacterial viability (data not shown). These results suggest that divalent and trivalent cations are likely involved in antibacterial activity of both mineral products, however, the change in pH with addition of EDTA is a critical factor, making the role of EDTA chelation unclear.

The pH of untreated BY07 and CB07 10% mineral suspensions and leachates was notably lower than pH 7.0, which is the typical pH for *E. coli* growth media (Table 1). When the pH of each mineral leachate was adjusted to pH 7.0, using 1 M sodium hydroxide, bactericidal activity was eliminated (data not shown), demonstrating an important role for low pH conditions in antibacterial activity. In addition, suspensions of minerals pre-washed with EDTA and leachate solutions containing 10 mM EDTA, which did not demonstrate antibacterial activity, had elevated pH levels relative to suspensions of minerals washed with water and mineral suspensions and leachate solutions without addition of EDTA, which retained bactericidal activity (Table 1). Adjusting the pH of leachate solutions with 10 mM EDTA back to the original leachate pH restored antibacterial activity of BY07 leachate, but reduced the rate of antibacterial activity of CB07 leachate (Figure 4). While CB07 leachate alone at pH 3.4 completely killed *E. coli* after 2 h of incubation, CB07 leachate with 10 mM EDTA at pH 3.4 resulted in only a 3-log_10_ reduction in viable cells (Figure 4). The enhanced survival of *E. coli* in CB07 leachate with EDTA for 2 h, despite the adjusted low pH, suggests that chelated cations are involved in CB07 toxicity. In contrast, BY07 leachate toxicity was not affected by EDTA addition when the low pH was maintained (Figure 4). Therefore, BY07 leachate toxicity is more directly dependent on pH and/or is dependent on cations that are not avidly chelated by EDTA. Treated and washed minerals and leachates with pH 5.3 or higher did not demonstrate antibacterial activity in 24 h, pointing to a critical change in the chemical environment between pH 5.3 and pH 5.7 for mineral suspensions and between pH 4.2 and 5.4 for leachates.

**Table 1:** Bactericidal activity and pH comparison of mineral suspensions, mineral leachates, and sodium phosphate buffers.

BY07 Preparation	pH
Leachate^a^	3.5 – 3.7*
Leachate +10 mM EDTA	6.2
10% mineral suspension	3.5*
10% water-treated mineral suspension	3.8*
10% EDTA-treated mineral suspension	5.7
10% 2,2′-bipyridyl-treated mineral suspension	4.1*

CB07 Preparation	pH
Leachate^a^	3.3 – 3.4*
Leachate +10 mM EDTA	5.5
10% mineral suspension	3.3*
10% water-treated mineral suspension	3.4*
10% EDTA-treated mineral suspension	5.3*
10% 2,2′-bipyridyl-treated mineral suspension	3.7*

Buffer Preparation	pH
100 mM phosphate buffer	3.7*
100 mM phosphate buffer	3.4*

a The pH of mineral leachate preparations varied slightly.

*Indicates bactericidal activity within 24 h.

To clarify the effects of pH alone on *E. coli* cell death, time course killing experiments were performed, revealing that the BY07 leachate resulted in a continuous decline in CFU. The greatest reduction occurred in the first 3 h of incubation in leachate. The viability of *E. coli* (initial concentration of 10^7^ CFU/mL) was reduced by >99.9% within 6 h and demonstrated complete killing at 24 h when incubated in BY07 leachate. Incubation in phosphate buffer at pH 3.7 resulted in a much more gradual decline in survival with a less than 2-log_10_ reduction in CFU at 6 h and survival of >10^4^ CFU/mL after 24 h (Figure 2). CB07 leachate completely eliminated survival of 10^7^ CFU/mL of *E. coli* within 1 h, while sodium phosphate buffer treatment at pH 3.4 reduced CFU/mL only 1-log_10_ in 1 h (Figure 2). Because *E. coli* viability was reduced significantly more rapidly by leachate solutions than by buffers at the same pH, we conclude that the low pH alone does not completely explain the antibacterial effects of these mineral products.

In addition, we specifically targeted the potential involvement of ferrous iron by pre-washing of the BY07 and CB07 minerals with the ferrous iron-specific chelator, 2,2′-bipyridyl. Kohanski et al. [27] recently reported that three bactericidal antibiotics, each with a different mechanism of action, induced *E. coli* and *S. aureus* cell death by hydroxyl radical formation within cells via Fenton reactions. Bactericidal effects of the antibiotics were prevented by addition of the ferrous iron chelator, 2,2′-bipyridyl, or the hydroxyl radical scavenger, thiourea, to the media [27]. BY07 and CB07 minerals pre-washed with 5 mM 2,2′-bipyridyl for 2 h showed no reduction in antimicrobial activity against *E. coli* (Figure 3). Furthermore, mineral leachate solutions treated with the addition of 500 µM or 1 mM 2,2′-bipyridyl showed no change in antibacterial activity (data not shown) confirming that elevated levels of ferrous iron are not independently responsible for mineral and leachate toxicity.

**Figure 5:**
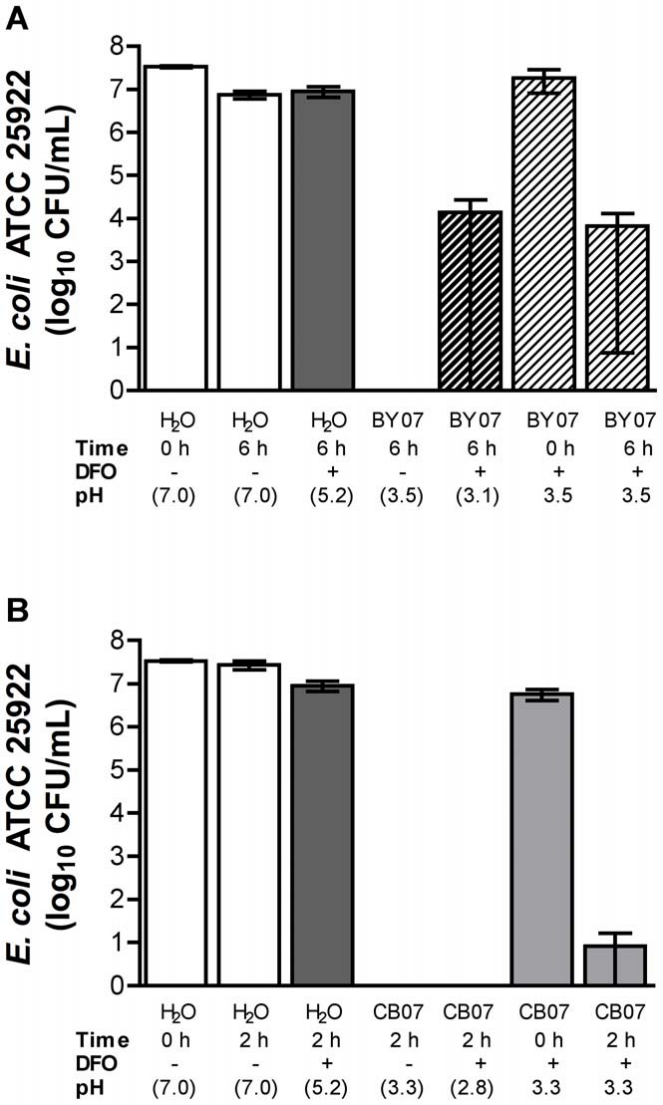
*E. coli* survival in 10% mineral suspensions with addition of 5 mM desferrioxamine. *E. coli* survival in 10% BY07 (A) and CB07 (B) mineral suspensions (100 mg/mL in dH_2_O) for 6 h and 2 h, respectively, with and without addition of 5 mM desferrioxamine (DFO) and pH adjustment. pH values shown within parentheses represent unadjusted pH readings. Values represent the mean CFU and standard error of at least three independent experiments.

To assess the potential contribution of ferric iron in antibacterial activity, desferrioxamine (5 mM final concentration), a strong ferric iron chelator [28], was added to 10% mineral suspensions of BY07 and CB07. The three hydroxamic acid groups within desferrioxamine allow for very tight chelation of ferric ions (logK_f_ stability constant of 31). In addition to Fe^3+^, desferrioxamine also forms stable complexes with Cu^2+^, Co^2+^, Zn^2+^, Fe^2+^, and Ni^2+^ with logK_f_ values of 14, 11, 11, 10, and 10, respectively [28]. In control experiments, *E. coli* incubated 6 h in pH 3.5 buffer supplemented with 0.11 mM FeCl_3_ significantly reduced CFU relative to *E. coli* in pH 3.5 buffer alone, demonstrating the potential toxicity of ferric iron at pH 3.5 (Figure S1). While a 10% BY07 mineral suspension completely killed *E. coli* within 6 h, incubation of *E. coli* with a desferrioxamine-treated 10% BY07 mineral suspension resulted in an average survival of 1.4×10^4^ CFU/ml in triplicate experiments, with the large error bar reflecting complete bacterial killing observed in two independent experiments (Figure 5A). Addition of desferrioxamine to the BY07 mineral suspension caused the pH to decrease to 3.1, therefore, we adjusted the pH of desferrioxamine-treated BY07 pH to 3.5 (pH of 10% BY07 suspension). Incubation of *E. coli* and pH-adjusted, desferrioxamine-treated BY07 resulted in an average survival of 6.5×10^3^ CFU/ml with complete killing observed in a single experiment (Figure 5A). These results suggest that both pH and ferric iron (or other metal cations sufficiently sequestered by desferrioxamine) contribute to BY07 mineral bacterial toxicity. Incubation of the desferrioxamine-treated CB07 mineral suspension with *E. coli* resulted in complete bactericidal activity within 2 h (Figure 5B). Since addition of desferrioxamine to the CB07 mineral suspension caused the pH to decrease to 2.8, the pH of desferrioxamine-treated CB07 was adjusted to pH to 3.3 (pH of 10% CB07 suspension). Incubation of *E. coli* and pH-adjusted, desferrioxamine-treated CB07 resulted in nearly complete bacterial killing in triplicate experiments (Figure 5B), suggesting that ferric iron and other metal cations mentioned above are not mediating CB07-induced toxicity or that the concentration of DFO was insufficient to prevent toxicity. Although iron is the primary metal species involved in hydroxyl radical formation, other transition metals known to have toxic effects on bacteria, such as copper, manganese, chromium, cobalt, and zinc, can also catalyze Fenton-like reactions by cycling between reduced and oxidized forms [29]. Accumulation of oxidative radical species, generated by Fenton and Fenton-like reactions, results in DNA damage [11], lipid peroxidation [30], and depletion of sulfhydryls [31].

To further assess the potential role of Fenton-like reactions in antibacterial activity, bathocuproine chelation and thiourea supplementation experiments were performed. Bathocuproine forms complexes with Cu^1+^, Cu^2+^, and Zn with stability constants (logK_f_) of 20, 7.5, and 4, respectively [32]. Addition of 5 mM bathocuproine (final concentration) to a 10% suspension of BY07 or CB07 mineral mixture immediately prior to contact with *E. coli* did not alter the antibacterial activity of the mineral suspension and resulted in complete killing of *E. coli* within 6 h or 2 h, respectively (data not shown). These results suggest that decreased bacterial viability is not a result of copper and/or zinc toxicity. When 150 mM thiourea was added to the BY07 and CB07 aqueous leachates or water immediately prior to contact with *E. coli*, antibacterial activity remained unchanged, resulting in complete killing of *E. coli* within 24 h (data not shown). These results provide strong evidence that the antibacterial mechanism of action for both mineral mixtures is independent of hydroxyl radical formation.

### NMR and ICP-OES Determination of Metal Accessibility and Concentration

We have investigated the accessibility of paramagnetic metal species in leachate samples by determining the effects of the leachates on the ^19^F NMR spectra of trifluoroacetic acid and fluoride ions. Transverse relaxation of NMR signals can be characterized by either the relaxation time (T_2_) or the relaxation rate (R_2_ = 1/T_2_). Increases in NMR relaxation rates are commonly seen in solutions containing paramagnetic metal ions. Paramagnetic relaxation enhancement results from interactions of the observed nucleus with the unpaired electron on the metal ion. These interactions can occur in solvent-separated ion pairs as well as when the species containing the observed nucleus is coordinated to the metal ion. Swift and Connick [33] first described the effects of dynamic exchange of an observed nucleus between a coordination complex and solvent-separated ion pairs. We have observed previously that the ^19^F resonance of fluoride ion, but not that of trifluoroacetic acid, is strongly affected by transient coordination to paramagnetic metal ions [34]. Metal ions that are not accessible to coordination by fluoride do not enhance the rate of relaxation of the fluoride resonance. By measuring the effects of leachates on the ^19^F resonances of fluoride and trifluoroacetic acid, we could determine whether metal ions in the leachate were chemically accessible to solution.

In our experiments, transverse relaxation rates of the trifluoroacetic acid resonance were not significantly affected by any of the leachate samples (data not shown), indicating that dipolar interactions of solvent separated species were insufficient to induce rapid NMR relaxation. Similarly, leachates from non-antibacterial mineral samples (CJ08 and DT05) had little effect on the fluoride ion ^19^F NMR relaxation rate (Table S3), indicating that paramagnetic metal species in non-antibacterial mineral leachates were not accessible for chemical coordination. In contrast, relaxation of the fluoride resonance was dramatically accelerated in leachates from the BY07 and CB07 mineral mixtures (Table S3). Since interactions between the solvent-separated paramagnetic metal ions and the fluoride ion should be similar to those of the metal ion with the trifluoroacetic acid ion, the enhancement of the fluoride ^19^F relaxation rate can be attributed to the effects of chemical coordination of the fluoride ion to the paramagnetic metal. The specificity of the effect indicates that the metal ions in the leachates from the BY07 and CB07 mineral mixtures are accessible to solution, but those in the non-bactericidal leachates are not. We note that total iron content (as determined by ICP-OES) did not correlate with bactericidal activities of the leachates, as BY07 had less total iron than non-bactericidal leachates, while CB07 had more (Table S3). In contract, the two bactericidal leachates had more total copper than either of the two non-antibacterial leachates (Table S3).

Experiments intended to determine the effects of pH on metal accessibility were complicated by protonation of the probe ion, fluoride, at a pH close to that of the leachate solutions (the pK_a_ of hydrofluoric acid is 3.20). We have previously reported that fluoride relaxation by FeEDTA− is greatly accelerated at pH where hydrofluoric acid concentrations are significant [34]. To avoid ambiguities arising from fluoride protonation, we measured the effects of the mineral leachates in buffered solutions ranging in pH from 5.0 to 9.0. Fluoride relaxation rates in the buffered solutions were significantly lower than in the more acidic, unbuffered solutions. In addition, the relaxation enhancements observed in a buffered solution decreased with time, eventually reaching what appeared to be an equilibrium value (data not shown). These observations suggest a slow change in the speciation of the metal complex occurs upon changing the pH.

## Discussion

The *in vitro* antibacterial activity assays presented here demonstrate the strong bactericidal action of BY07 and CB07 products. In particular, MRSA and ESBL *E. coli* susceptibility to BY07 and CB07 highlights the potential for applications of hydrated mineral products or derivatives as complementary therapeutics in cases of antibiotic-resistant topical infections. The antibacterial activity of mineral leachates demonstrated that the chemical properties of the minerals, rather than physical forces, are involved in antibacterial activity. These results are consistent with the observations of Haydel et al. [17] who reported loss of antibacterial activity in minerals that had been chemically altered by cation exchange, but had not been structurally altered.

Parallel *E. coli* survival curves in phosphate buffer at the same pH as mineral leachates demonstrated that pH stress is not the only chemical factor influencing bacteria survival. As an enteric pathogen, *E. coli* demonstrates a high level of acid tolerance, which facilitates survival in the low pH environment of the digestive tract [35]. *E. coli* has been shown to maintain growth at a pH as low as 4.7 in minimal media and can maintain an internal pH up to 2 units higher than the environment [36]. The role of pH in antibacterial activity of BY07 and CB07 is likely related to the effect of pH on toxicity of metal ions by influencing chemical speciation and bioavailability [37]. Bioavailability of free metal ions in soil is dependent on cation exchange capacity of the mineral surfaces, ability to form carbonate, sulfide, or hydroxide complexes, precipitation, and adsorption, all of which are influenced by both pH and ion concentration [38], [39].

With the exception of arsenic, molybdenum, and selenium, metal ion solubility increases as pH decreases [38]. Free, reactive, metal ions may be limited in solution by hydroxide precipitation or adsorption at higher pH values. Co^3+^, Ti^4+^, and Fe^3+^ hydroxides precipitate above pH 2.5 at 25°C [39] and therefore, are unlikely to be present in BY07 and CB07 leachate solutions with pHs of 3.7 and 3.4, respectively. Al^3+^, Cr^3+^, Be^2+^, and Cu^2+^ precipitate between pH 3.0 and 6.0 [10], [39], which could explain loss of toxicity of leachate and mineral suspensions at pH>4.5 – 5.5.

While no single metal species has been identified as an independent antibacterial factor, there is a correlation between antibacterial activity and accessibility of metal ions in leachate solutions, particularly elevated iron concentrations. CB07 antibacterial activity occurs more rapidly than that of BY07 (Figure 2), possibly due to the significantly higher level of chemically-available iron in CB07 as indicated by NMR relaxation rates (Table S3). Only the broad-spectrum chelator, EDTA, affected the bactericidal activity of CB07 when a low pH was maintained, suggesting that a combination of cations may be involved in CB07 leachate toxicity. However, EDTA chelation efficiency is progressively reduced as the carboxyl groups become protonated at pH levels below the pKa values of 10.2, 6.2, 2.7, and 2.0, and therefore the role of pH and chelation still cannot be assessed independently. Reduction of BY07 antibacterial activity in the presence of desferrioxamine, but not bathocuproine, suggests that Fe^3+^, Co^2+^, and Ni^2+^, which are avidly chelated by desferrioxamine, but not by bathocuproine, are the most likely metal ions involved in the antibacterial activity of BY07.

Experiments focused on assessing cellular and/or physiological damage to *E. coli* cells treated with the mineral leachates should also be pursued. Co^2+^ and Ni^2+^ demonstrate antibacterial activity by inhibiting DNA replication [40] and reducing RNA and protein synthesis [41], therefore these cellular activities should be assessed. Furthermore, identifying *E. coli* strains resistant to mineral treatments and characterizing changes in gene expression may provide further insight to the bactericidal mechanism of action of these mineral products.

Because the low pH environment appears critical to antibacterial activity of CB07 and BY07, efficacy may be reduced in a topical application in which the pH of the tissues treated is significantly higher than the mineral suspensions. Sustained contact with low pH mineral products may also result in damage to tissues, making direct topical application infeasible. *In vivo* studies will be essential to determining applications in which hydrated mineral products can be effectively and safely used.

An additional challenge of applying natural mineral products in therapeutic processes is consistency in the mineral mixtures. Natural minerals are heterogeneous mixtures that can vary remarkably within a small geographical area. Thus, it is essential to understand the specific characteristics needed for antibacterial activity in order to validate use, improve quality control of mineral and chemical consistency, and facilitate rapid, reliable identification of bactericidal mineral mixtures.

## Materials and Methods

### Bacterial Strains and Growth Conditions

*E. coli* ATCC 25922, ESBL *E. coli* ATCC 51446, *S.* Typhimurium ATCC 14028, *P. aeruginosa* ATCC 27853, and *S. aureus* ATCC 29213 are clinical isolates obtained from the American Type Culture Collection. The MRSA isolate was obtained from Sonora Quest Laboratories (Tempe, AZ) [17]. Antibiotic resistance profiles of the ESBL *E. coli* and MRSA strains were previously described by Haydel et al. [17] *E. coli* was cultured using Luria-Bertani (LB) broth or agar, *S.* Typhimurium was cultured using nutrient broth or agar, and *S. aureus*, MRSA, and *P. aeruginosa* strains were cultured in trypticase soy broth or agar. All strains were grown at 37°C with gentle rotary mixing to prevent sedimentation and to ensure continuous contact with minerals.

### Mineral Mixtures and Mineral Mixture Leachates

The BY07 and CB07 mineral mixtures were obtained from commercial suppliers and were sterilized by autoclaving for 1 h at 121°C before use. Mineral suspensions refer to sterilized mineral mixtures suspended in sterile ultra-pure, deionized H_2_O. Mineral leachate solutions were prepared by stirring mineral suspensions in UV-irradiated, ultra-pure deionized H_2_O (1 g per 20 mL) for 18–24 h at room temperature to allow for sufficient mineral hydration and chemical equilibration with the fluid. The insoluble minerals were then removed from the suspension by centrifugation for 3 h at >31,000×*g*, and the supernatant (mineral leachate) was sterilized by passage through a 0.22 µm filter before subsequent use.

### Broad-Spectrum Antibacterial Assays

Minerals were tested for antibacterial activity as previously described [17]. Briefly, overnight cultures of bacterial strains were diluted in fresh medium to ∼10^7^ CFU/400 µL. Initial bacterial counts were confirmed by plating serially diluted bacterial cultures on appropriate agar plates and enumerating colonies after 24 h incubation at 37°C. Sterilized minerals (200 mg) were suspended in 400 µL of the initial bacterial suspensions. Positive controls of bacteria without minerals were included in each experiment. To confirm that clays remained sterile during storage after initial autoclaving, negative controls of minerals in LB without bacteria were also included periodically. After 24 h incubation, 10-fold serial dilutions were plated on LB agar and enumerated. In addition, 100 µL of undiluted mineral-bacteria suspensions were plated directly on LB agar.

### Antibacterial Susceptibility Testing of Mineral Leachates and 10% Mineral Suspensions

*E. coli* ATCC 25922 from overnight cultures were diluted in fresh LB and grown as early logarithmic phase cultures. Bacterial cells were collected by centrifugation, rinsed once in 0.1X phosphate-buffered saline (PBS), and suspended in the appropriate leachate solution, 10% mineral suspension, or sterile H_2_O at an initial concentration of ∼10^7^ CFU/mL. Initial CFU concentrations were confirmed by plating of the control bacterial population on LB agar and enumerating colonies after 24 h incubation at 37°C. Experimental samples were incubated at 37°C with gentle rotary mixing, and *E. coli* survival was determined by plating duplicate 10-fold serial dilutions in sterile H_2_O for each sample at appropriate time points and enumerating colonies on plates after 24 h incubation at 37°C.

### Preparation of EDTA- and 2,2′-Bipyridyl-Treated Mineral Mixtures

BY07 and CB07 mineral mixtures were pre-washed as suspensions of 1 g per 20 mL in either 5 mM aqueous 2,2′-bipyridyl (final concentration), 100 mM aqueous EDTA-2Na·2H_2_O (final concentration), or deionized H_2_O. To ensure full equilibration of minerals and the specific chelator, pre-washing was maintained in EDTA-2Na·2H_2_O for 2.5 h with agitation during the first 30 min [42], in 2,2′-bipyridyl for 2 h with continuous agitation, and in deionized H_2_O for 48 h without agitation. After two washes in deionized H_2_O to remove residual chelator, minerals were recovered by centrifugation, (2 h at >31,000×*g*), dried, crushed with a mortar and pestle, and sterilized by autoclaving for 1 h at 121°C. 10% suspensions (in sterile, deionized H_2_O) of each treated mineral product were tested for antibacterial activity.

### Mineral Leachate Chelation Experiments

EDTA-2Na·2H_2_O and 2,2′-bipyridyl were added directly to the aqueous mineral leachates at final concentrations of 10 mM and 1 mM, respectively. The pH of each chelator-adjusted leachate solution was measured and subsequently tested for antibacterial activity against *E. coli* ATCC 25922. In addition, the BY07 and CB07 leachates containing 10 mM EDTA-2Na·2H_2_O were subsequently adjusted with 1 M HCl to generate a pH environment similar to the initial acidic pH of each mineral leachate. EDTA-, 2,2′-bipyridyl-, and pH-adjusted BY07 and CB07 mineral leachates were tested for antibacterial activity against *E. coli* ATCC 25922. A control of 0.11 mM FeCl_3_ (final concentration) at pH 3.5 was also tested for antibacterial activity against *E. coli* ATCC 25922 to determine the toxicity of iron at a concentration equal to the level of iron in BY07 leachate. To assess the kinetics of *E. coli* survival in a controlled pH environment similar to that of the BY07 and CB07 mineral leachates, sodium phosphate buffer, equilibrated to pH 3.7 and pH 3.4, respectively, was used. Early logarithmic phase *E. coli* were suspended in mineral leachate, 100 mM sodium phosphate buffer (pH 3.7 or pH 3.4), or deionized H_2_O to an initial concentration of ∼10^7^ CFU/mL and incubated with rotary mixing for 24 h at 37°C. At appropriate time points, 100 µL aliquots of each sample were serially diluted and plated on LB agar plates. Bacterial viability was assessed after the plates were incubated for 24 h at 37°C.

### 10% Mineral Suspension Chelation and Thiourea Supplementation

5 mM desferrioxamine (final concentration) or 5 mM bathocuproine (final concentration) was added directly to 10% BY07 or CB07 mineral suspensions. The pH of each chelator-adjusted mineral suspension was measured, and the suspensions were tested for antibacterial activity against *E. coli* ATCC 25922 as described above. In addition, the BY07 and CB07 mineral suspensions containing 5 mM desferrioxamine were subsequently adjusted with 1 M sodium phosphate buffer to generate a pH environment similar to the initial acidic pH of the 10% mineral suspensions. pH-adjusted mineral-chelator suspensions were then tested for antibacterial activity against *E. coli* as described above. To investigate the potential role of hydroxyl radical formation in antimicrobial activity, 10% mineral mixture suspensions with addition of 150 mM aqueous thiourea (final concentration) were also tested for antibacterial activity.

### X-ray Diffraction (XRD)

Qualitative and quantitative XRD analyses of mineral mixtures were performed by CAMET Research, Inc. (Goleta, CA) and collected on a horizontal wide angle powder diffractometer (Rigaku, Japan) with a graphite monochromator in the diffracted beam path utilizing Cu-Kα radiation. For qualitative analyses, the minerals were carefully ground with a mortar and pestle and mounted in a cavity sample holder. Qualitative XRD data was collected from 2°–65° 2-theta with step-size increments of 0.025° and a count time of 1 second per step. For quantitative analyses, the minerals and 10% ZnO (NIST SRM 674a) as an internal standard were mixed and pulverized in ethanol using a ball-type SPEX mill, dried at 65°C, and ground with a mortar and pestle. Quantitative XRD data was collected from 2°–65° 2-theta with step-size increments of 0.02° and a count time of 2 seconds per step. The utilized data range for the quantitative XRD analysis with RockJock [43] was from 20°–65° 2-theta.

### X-ray Fluorescence (XRF)

Major elemental analyses of mineral mixtures were performed by CAMET Research, Inc. (Goleta, CA) and collected on a Siemens SRS 200 wavelength dispersive X-ray fluorescence (WDXRF) spectrometer. Calibration curves for the various oxides were established with USGS rocks (AGV-2, BCR-2 and GSP-2), Estonia rocks (ES-3, ES-4 and ES-14) [44], and reagent grade calcium carbonate. Approximately 1.5–3 g of material was placed in a ceramic crucible, dried, and calcined at 110°C, 550°C, and 950°C in a muffle furnace. The calcined material was mixed with a lithium borate flux and fused at 1000°C. The finished beads were polished on a diamond wheel to produce a flat surface for analysis.

### Nuclear Magnetic Resonance (NMR)

Samples for the initial NMR experiments were prepared by mixing equal amounts of the mineral leachate and a trifluoroacetic acid/fluoride-containing solution. Transverse relaxation rates were determined for the ^19^F resonances of the two additives using the standard Carr-Purcell-Meiboom-Gill (CPMG) method [45] and a JEOL Eclipse 300 MHz NMR spectrometer. NMR relaxation of the fluoride nucleus in solutions containing leachates from samples BY07 and CB07 were too rapid to measure at this concentration. Therefore, reported relaxation rates were determined from diluted samples by extrapolation back to full concentration.

### Inductively Coupled Plasma Optical Emission Spectrometry (ICP-OES)

Concentrations of copper and iron in the mineral leachates were determined by ICP-OES using a PerkinElmer Optima 4100 spectrometer. Samples for ICP-OES analysis were prepared by the addition of 40 µL of concentrated nitric acid to 10 mL aliquots of the leachate samples described above. Calibration curves were prepared using known concentrations of either copper (ranging from 0.5 to 160 ppb) or iron (ranging from 1 to 10 ppm). For each of the leachate samples, emission spectra were recorded for copper solutions at 324, 224, and 327 nm, and iron emission intensities were recorded at 238, 259, and 273 nm. With the exception of the BY07 mineral leachate, concentrations of iron were too high for accurate ICP-OES analysis from the leachates themselves. For these samples, each leachate was diluted 100∶1 with a concentrated nitric acid solution, and the iron in the diluted samples was determined from the emission intensities.

## Supporting Information

Figure S1 *E. coli* survival in low pH phosphate buffer supplemented with FeCl_3_. *E. coli* survival in pH 3.5 sodium phosphate buffer with and without FeCl_3_ (0.11 mM final concentration) supplementation for 6 h. Values represent the mean CFU and standard error of three independent experiments.
(0.44 MB TIF)

Table S1 Mineral composition of BY07 and CB07 mineral mixtures.
(0.03 MB DOC)

Table S2 Major chemical composition of BY07 and CB07 mineral mixtures with and without pre-washing with 100 mM EDTA.
(0.04 MB DOC)

Table S3 Chemical composition of leachates and fluoride ^19^F NMR relaxation rates.
(0.03 MB DOC)
