# Supplementary material for: pH-Dependent Metal Ion Toxicity Influences the Antibacterial Activity of Two Natural Mineral Mixtures
Source: PLoS One. 2010 Mar 1;5(3):e9456. doi: 10.1371/journal.pone.0009456 (PMC2830476; doi:10.1371/journal.pone.0009456)
Supplement: Table S1 — Mineral composition of BY07 and CB07 mineral mixtures. (0.03 MB DOC) [file pone.0009456.s002.doc]

| **Mineral content** | **BY07(wt%)** | **CB07(wt%)** |
| --- | --- | --- |
| Quartz | 13.70 | 45.50 |
| Anorthoclase feldspar | 23.00 | 1.30 |
| Gypsum | 0.70 | 2.20 |
| Pyrite | 0.00 | 1.90 |
| Jarosite | 7.60 | 2.60 |
| **Total non-clays** | **45.10** | **53.50** |
|  |  |  |
| Ca-smectite (Wyo) | 37.30 | 17.20 |
| Ferruginous smectite | 0.00 | 4.20 |
| 1M illite (R>1, 70-80%I) | 7.90 | 19.80 |
| Chlorite (Tusc) | 9.70 | 5.20 |
| **Total clays** | **54.90** | **46.40** |
