# Supplementary material for: pH-Dependent Metal Ion Toxicity Influences the Antibacterial Activity of Two Natural Mineral Mixtures
Source: PLoS One. 2010 Mar 1;5(3):e9456. doi: 10.1371/journal.pone.0009456 (PMC2830476; doi:10.1371/journal.pone.0009456)
Supplement: Table S2 — Major chemical composition of BY07 and CB07 mineral mixtures with and without pre-washing with 100 mM EDTA. (0.04 MB DOC) [file pone.0009456.s003.doc]

| **[Chemical composition](../Manuscript_Physiochemical%20properties/manuscript%20Tables.xls#'Chemical%20composition'!B2)** | **[BY07 (wt%)](../Manuscript_Physiochemical%20properties/manuscript%20Tables.xls#'Chemical%20composition'!B2)** | **[EDTA-washed BY07 (wt%)](../Manuscript_Physiochemical%20properties/manuscript%20Tables.xls#'Chemical%20composition'!B2)** | **[CB07 (wt%)](../Manuscript_Physiochemical%20properties/manuscript%20Tables.xls#'Chemical%20composition'!B2)** | **[EDTA-washed CB07 (wt%)](../Manuscript_Physiochemical%20properties/manuscript%20Tables.xls#'Chemical%20composition'!B2)** |
| --- | --- | --- | --- | --- |
| [SiO](../Manuscript_Physiochemical%20properties/manuscript%20Tables.xls#'Chemical%20composition'!B2)_2_ | [53.48](../Manuscript_Physiochemical%20properties/manuscript%20Tables.xls#'Chemical%20composition'!B2) | [53.64](../Manuscript_Physiochemical%20properties/manuscript%20Tables.xls#'Chemical%20composition'!B2) | [65.74](../Manuscript_Physiochemical%20properties/manuscript%20Tables.xls#'Chemical%20composition'!B2) | [68.01](../Manuscript_Physiochemical%20properties/manuscript%20Tables.xls#'Chemical%20composition'!B2) |
| [Al](../Manuscript_Physiochemical%20properties/manuscript%20Tables.xls#'Chemical%20composition'!B2)_2_O_3_ | [20.04](../Manuscript_Physiochemical%20properties/manuscript%20Tables.xls#'Chemical%20composition'!B2) | [20.27](../Manuscript_Physiochemical%20properties/manuscript%20Tables.xls#'Chemical%20composition'!B2) | [16.03](../Manuscript_Physiochemical%20properties/manuscript%20Tables.xls#'Chemical%20composition'!B2) | [16.58](../Manuscript_Physiochemical%20properties/manuscript%20Tables.xls#'Chemical%20composition'!B2) |
| [Fe](../Manuscript_Physiochemical%20properties/manuscript%20Tables.xls#'Chemical%20composition'!B2)_2_O_3_ | [8.00](../Manuscript_Physiochemical%20properties/manuscript%20Tables.xls#'Chemical%20composition'!B2) | [8.88](../Manuscript_Physiochemical%20properties/manuscript%20Tables.xls#'Chemical%20composition'!B2) | [4.42](../Manuscript_Physiochemical%20properties/manuscript%20Tables.xls#'Chemical%20composition'!B2) | [4.16](../Manuscript_Physiochemical%20properties/manuscript%20Tables.xls#'Chemical%20composition'!B2) |
| [CaO](../Manuscript_Physiochemical%20properties/manuscript%20Tables.xls#'Chemical%20composition'!B2) | [2.06](../Manuscript_Physiochemical%20properties/manuscript%20Tables.xls#'Chemical%20composition'!B2) | [0.91](../Manuscript_Physiochemical%20properties/manuscript%20Tables.xls#'Chemical%20composition'!B2) | [1.62](../Manuscript_Physiochemical%20properties/manuscript%20Tables.xls#'Chemical%20composition'!B2) | [0.90](../Manuscript_Physiochemical%20properties/manuscript%20Tables.xls#'Chemical%20composition'!B2) |
| [MgO](../Manuscript_Physiochemical%20properties/manuscript%20Tables.xls#'Chemical%20composition'!B2) | [0.55](../Manuscript_Physiochemical%20properties/manuscript%20Tables.xls#'Chemical%20composition'!B2) | [0.52](../Manuscript_Physiochemical%20properties/manuscript%20Tables.xls#'Chemical%20composition'!B2) | [0.64](../Manuscript_Physiochemical%20properties/manuscript%20Tables.xls#'Chemical%20composition'!B2) | [0.56](../Manuscript_Physiochemical%20properties/manuscript%20Tables.xls#'Chemical%20composition'!B2) |
| [Na](../Manuscript_Physiochemical%20properties/manuscript%20Tables.xls#'Chemical%20composition'!B2)_2_O | [2.32](../Manuscript_Physiochemical%20properties/manuscript%20Tables.xls#'Chemical%20composition'!B2) | [3.63](../Manuscript_Physiochemical%20properties/manuscript%20Tables.xls#'Chemical%20composition'!B2) | [0.67](../Manuscript_Physiochemical%20properties/manuscript%20Tables.xls#'Chemical%20composition'!B2) | [1.33](../Manuscript_Physiochemical%20properties/manuscript%20Tables.xls#'Chemical%20composition'!B2) |
| [K](../Manuscript_Physiochemical%20properties/manuscript%20Tables.xls#'Chemical%20composition'!B2)_2_O | [1.50](../Manuscript_Physiochemical%20properties/manuscript%20Tables.xls#'Chemical%20composition'!B2) | [1.60](../Manuscript_Physiochemical%20properties/manuscript%20Tables.xls#'Chemical%20composition'!B2) | [1.89](../Manuscript_Physiochemical%20properties/manuscript%20Tables.xls#'Chemical%20composition'!B2) | [2.05](../Manuscript_Physiochemical%20properties/manuscript%20Tables.xls#'Chemical%20composition'!B2) |
| [TiO](../Manuscript_Physiochemical%20properties/manuscript%20Tables.xls#'Chemical%20composition'!B2)_2_ | [0.82](../Manuscript_Physiochemical%20properties/manuscript%20Tables.xls#'Chemical%20composition'!B2) | [0.82](../Manuscript_Physiochemical%20properties/manuscript%20Tables.xls#'Chemical%20composition'!B2) | [0.62](../Manuscript_Physiochemical%20properties/manuscript%20Tables.xls#'Chemical%20composition'!B2) | [0.66](../Manuscript_Physiochemical%20properties/manuscript%20Tables.xls#'Chemical%20composition'!B2) |
| [P](../Manuscript_Physiochemical%20properties/manuscript%20Tables.xls#'Chemical%20composition'!B2)_2_O_5_ | [0.23](../Manuscript_Physiochemical%20properties/manuscript%20Tables.xls#'Chemical%20composition'!B2) | [0.24](../Manuscript_Physiochemical%20properties/manuscript%20Tables.xls#'Chemical%20composition'!B2) | [0.10](../Manuscript_Physiochemical%20properties/manuscript%20Tables.xls#'Chemical%20composition'!B2) | [0.06](../Manuscript_Physiochemical%20properties/manuscript%20Tables.xls#'Chemical%20composition'!B2) |
| [SO](../Manuscript_Physiochemical%20properties/manuscript%20Tables.xls#'Chemical%20composition'!B2)_3_ | [1.64](../Manuscript_Physiochemical%20properties/manuscript%20Tables.xls#'Chemical%20composition'!B2) | [0.78](../Manuscript_Physiochemical%20properties/manuscript%20Tables.xls#'Chemical%20composition'!B2) | [1.18](../Manuscript_Physiochemical%20properties/manuscript%20Tables.xls#'Chemical%20composition'!B2) | [0.37](../Manuscript_Physiochemical%20properties/manuscript%20Tables.xls#'Chemical%20composition'!B2) |
|  |  |  |  |  |
| [LOI](../Manuscript_Physiochemical%20properties/manuscript%20Tables.xls#'Chemical%20composition'!B2)^a^ | [9.36](../Manuscript_Physiochemical%20properties/manuscript%20Tables.xls#'Chemical%20composition'!B2) | [8.70](../Manuscript_Physiochemical%20properties/manuscript%20Tables.xls#'Chemical%20composition'!B2) | [7.09](../Manuscript_Physiochemical%20properties/manuscript%20Tables.xls#'Chemical%20composition'!B2) | [8.70](../Manuscript_Physiochemical%20properties/manuscript%20Tables.xls#'Chemical%20composition'!B2) |
|  |  |  |  |  |
| [Total](../Manuscript_Physiochemical%20properties/manuscript%20Tables.xls#'Chemical%20composition'!B2) | [100](../Manuscript_Physiochemical%20properties/manuscript%20Tables.xls#'Chemical%20composition'!B2) | [100](../Manuscript_Physiochemical%20properties/manuscript%20Tables.xls#'Chemical%20composition'!B2) | [100](../Manuscript_Physiochemical%20properties/manuscript%20Tables.xls#'Chemical%20composition'!B2) | [100](../Manuscript_Physiochemical%20properties/manuscript%20Tables.xls#'Chemical%20composition'!B2) |

[a](../Manuscript_Physiochemical%20properties/manuscript%20Tables.xls#'Chemical%20composition'!B2)[Loss on ignition.](../Manuscript_Physiochemical%20properties/manuscript%20Tables.xls#'Chemical%20composition'!B2)
