# Supplementary material for: pH-Dependent Metal Ion Toxicity Influences the Antibacterial Activity of Two Natural Mineral Mixtures
Source: PLoS One. 2010 Mar 1;5(3):e9456. doi: 10.1371/journal.pone.0009456 (PMC2830476; doi:10.1371/journal.pone.0009456)
Supplement: Table S3 — Chemical composition of leachates and fluoride 19F NMR relaxation rates. (0.03 MB DOC) [file pone.0009456.s004.doc]

| **Mineral sample** | **pH^1^** | **R_2,f_ (s^-1^)** | **[Cu] uM^2^** | **[Fe] mM^2^** |
| --- | --- | --- | --- | --- |
| None |  | 1.4 | 0 | 0 |
| CJ08 | 7.77 | 4.6 | 0.031 | 3.31 |
| DT05 | 6.13 | 3.9 | 0.016 | 3.19 |
| BY07^3^ | 4.35 | 644 | 0.173 | 0.11 |
| CB07^3^ | 4.35 | 1750 | 0.394 | 11.22 |
| ^1^pH measurements were performed using a 1:10 dilution of the unbuffered leachate in deionized H_2_O.  ^2^Metal ion concentrations were determined from ICP-OES analysis.  ^3^NMR relaxation rate for mineral leachates were determined by extrapolation of data measured for diluted samples. | | | | |
